# Association of US county-level social vulnerability index with breast, colorectal, and lung cancer screening, incidence, and mortality rates across US counties

**DOI:** 10.3389/fonc.2024.1422475

**Published:** 2024-08-07

**Authors:** Akhil Mehta, Won Jin Jeon, Gayathri Nagaraj

**Affiliations:** ^1^ Houston Methodist Dr. Mary and Ron Neal Cancer Center, Houston Methodist Hospital, Houston, TX, United States; ^2^ Division of Hematology and Medical Oncology, Loma Linda University Cancer Center, Loma Linda, CA, United States

**Keywords:** social vulnerability index, social determinants of health, colorectal cancer, breast cancer, lung cancer, cancer screening

## Abstract

**Background:**

Despite being the second leading cause of death in the United States, cancer disproportionately affects underserved communities due to multiple social factors like economic instability and limited healthcare access, leading to worse survival outcomes. This cross-sectional database study involves real-world data to explore the relationship between the Social Vulnerability Index (SVI), a measure of community resilience to disasters, and disparities in screening, incidence, and mortality rates of breast, colorectal, and lung cancer. The SVI encompasses four themes: socioeconomic status, household composition & disability, minority status & language, and housing type & transportation.

**Materials and methods:**

Using county-level data, this study compared cancer metrics in U.S. counties and the impact of high and low SVI. Two-sided statistical analysis was performed to compare SVI tertiles and cancer screening, incidence, and mortality rates. The outcomes were analyzed with logistic regression to determine the odds ratio of SVI counties having cancer metrics at or above the median.

**Results:**

Our study encompassed 3,132 United States counties. From publicly available SVI data, we demonstrated that high SVI scores correlate with low breast and colorectal cancer screening rates, along with high incidence and mortality rates for all three types of cancers. County level SVI has impact on incidence rates of cancers; breast cancer rates were lowest in high SVI counties, while colorectal and lung cancer rates were highest in the same counties. Age-adjusted mortality rates for all three cancers increased across SVI tertiles. After risk adjustment, a 10-point SVI increase correlated with lower screening and higher mortality rates.

**Conclusion:**

In conclusion, our study establishes a significant correlation between SVI and cancer metrics, highlighting the potential to identify marginalized communities with health disparities for targeted healthcare initiatives. It underscores the need for further longitudinal studies on bridging the gap in overall cancer care in the United States.

## 1 Introduction

Cancer ranks as the second leading cause of death in the United States (1). Despite an overall decline in cancer-related mortality nationwide, underserved communities with unfavorable social determinants of health (SDOH), such as economic instability, limited healthcare access, neighborhood deprivation, and racial/ethnic discrimination, continue to experience disproportionately high adverse cancer outcomes (2–4). Breast, colorectal, and lung cancer are among the most commonly diagnosed cancers in the country (5). Fortunately, effective screening methods like mammograms, colonoscopies, and low dose computed tomography scans have significantly reduced morbidity and mortality rates for these cancers (6, 7). However, underserved communities facing adverse SDOH often lack accessibility to these screening methods, exacerbating disparities in cancer outcomes (8). Consequently, it is crucial to assess the impact of SDOH on cancer screening, incidence, and mortality rates in the United States. To identify underserved communities that could benefit from targeted interventions to reduce overall cancer burden and improve patient outcomes (8).

The Social Vulnerability Index (SVI) developed by the Centers for Disease Control and Prevention/Agency for Toxic Substances and Disease Registry (CDC/ATSDR) is a novel, database that ranks each census tract (subdivisions of counties for which census collects statistical data) based on 16 social factors grouped into four themes—socioeconomic status, household composition & disability, minority status/language, and housing type and transportation—to gauge a community’s resilience in the face of external stressors such as natural disasters and infectious disease outbreaks (based on the 2018 edition) (9). SVI data is publicly available and is calculated by analyzing a 5-year US Census Bureau’s American Community Survey (ACS) data and using specialized geospatial mapping with the Geospatial Research, Analysis & Services Program (GRASP) (9). The CDC/ATSDR SVI values are typically categorized using a quantile classification (i.e. tertiles or quartiles etc.) and the classification goes from least vulnerable to most vulnerable, ranging from 0 to 1.0 (9). SVI plays a significant role in health services utilization and health-related outcomes, including but not limited to COVID-19 incidence and mortality, obesity, surgery utilization, and cancer screening by ranking counties based on the respective cumulative SVI levels (10–20). However, while previous studies have examined the relationship between disparities in cancer screening and SVI, none have thus far combinatorially explored the national-level association between disparities in cancer screening, incidence, and mortality rates with SVI. Thus, this ecologic study aimed to investigate the association between SVI at the county level in the United States and breast, colorectal, and lung cancer screening, incidence, and mortality rates.

## 2 Materials and methods

### 2.1 Cancer screening, incidence, and mortality data

County-level data on breast and colorectal cancer screening, incidence, and mortality rates were obtained from the State Cancer Profiles, which are made accessible through the National Cancer Institute (NIH) and the CDC (21). Similarly, county-level data on lung cancer incidence and mortality rates were also obtained from the same sources (21). Unfortunately, information on county-level lung cancer screening rates was not available for analysis. Breast cancer screening was defined as having a mammogram within the past two years among women aged 40 years and older. For colorectal cancer screening, it was defined as having a sigmoidoscopy or colonoscopy among adults aged 50 years and older (22). The screening estimates were reported as proportion of eligible individuals within each US county who met the screening criteria. The most recent screening rate estimates were based on data from 2008 to 2010 and derived from a statistical model that combined information from the Behavioral Risk Factor Surveillance System (BRFSS) and the National Health Interview Survey (NHIS) (23, 24). Incidence and mortality rates for breast, colorectal, and lung cancer were reported by US county for all stages, covering the years 2013 to 2017, which represented the latest available 5-year average. These rates were age-adjusted and reported as cases per 100,000 individuals in the population (25–30). It is important to note that only females were included in the breast cancer data. These cancer outcomes were analyzed in tertiles along with SVI tertiles. To minimize selection bias, all the reported US counties with available screening rate estimates from the BRFSS and NHIS data were included. Counties without any estimates were removed during the ranking process.

### 2.2 Social vulnerability index data

The determination of overall social vulnerability in this study was based on the SVI, which encompasses four themes identified by the CDC/ATSDR according to the 2018 edition (9). The SVI scores range from 0 to 1, with higher scores indicating greater vulnerability. Besides the numeric SVI, SVI data was categorized into tertiles for the purpose of the study and were analyzed as ordinal variables to explore the statistical significance with cancer outcomes in tertiles. Institutional review board approval was not required for this study, as it involved the analysis of publicly available, de-identified government-issued data that did not contain individually identifiable information.

### 2.3 Outcomes

The SVI tertiles were established based on the four themes of social factors comprising the 2018 SVI scores: socioeconomic status, household composition and disability, minority status and language, and housing type and transportation. The primary objective of this study was comparing screening, incidence, and mortality rates for breast, colorectal and lung cancer for high SVI US counties to low SVI US counties. For analysis, the primary outcome was defined as the odds ratio of screening rates being at or above the 50th percentile in high SVI US counties compared to low SVI US counties.

### 2.4 Statistical analysis/study design

The study was designed as a cross-sectional database study. Statistical significance between groups was assessed at an alpha level of less than 0.05. All analyses were performed using Stata 16 (StataCorp LLC, College Station, TX, USA). All tests were two-sided, and significance was determined with a p-value less than 0.05. Logistic regression analysis was used to obtain the odds ratios for cancer screening rates, with the dependent variable being the screening rate at or above the 50th percentile. The independent variable was the SVI tertile, adjusted for potential confounders, namely age. Screening, incidence, and mortality rates were adjusted for age, but other demographic characteristics were not adjusted for. Therefore, residual confounding attributable to these variables could not be accounted for. For the comparison of cancer incidence and mortality rates among different SVI tertiles, ANOVA was employed to determine statistical differences across the groups. Bivariate choropleth maps were utilized to visually represent the correlation between SVI tertiles and cancer outcome tertiles.

## 3 Results

### 3.1 County characteristics relative to the social vulnerability index

**Figure 1:**
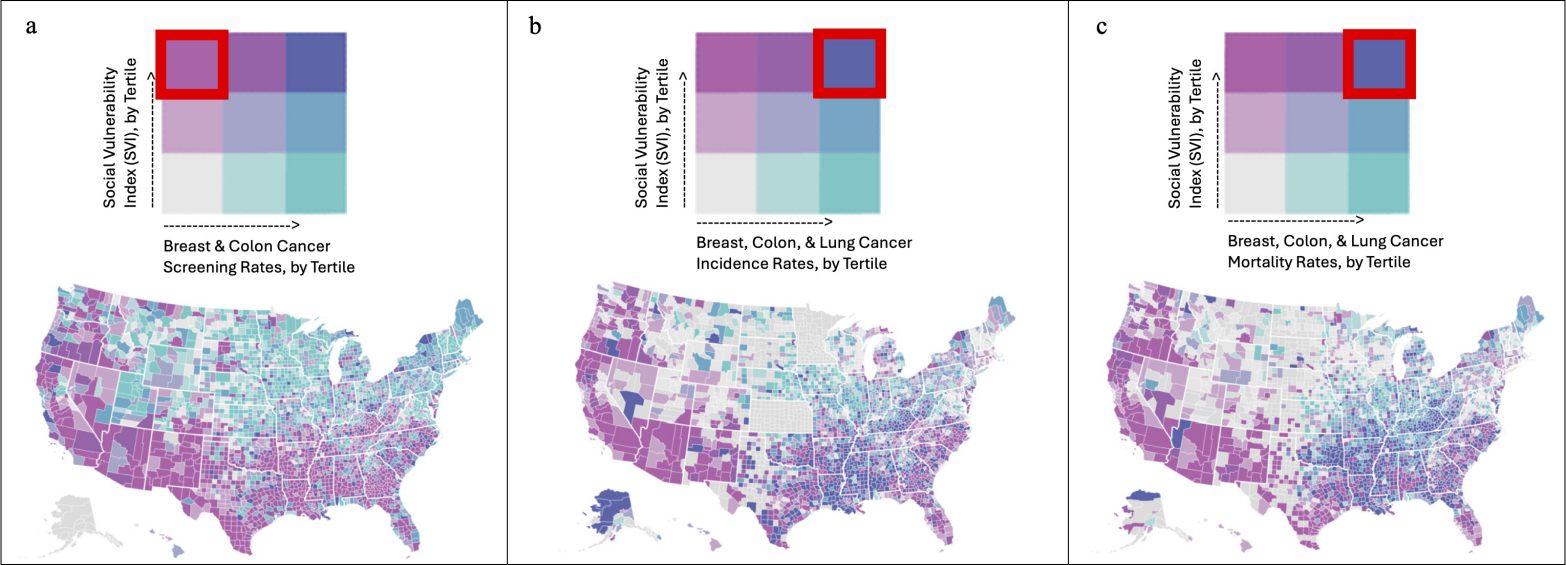
The southwestern and southeastern parts of the country exhibited the highest concentration of US counties with high SVI and low breast & colon cancer screening rates **(A)**. By contrast, the southeastern parts of the country exhibited the highest concentration of US counties with high SVI and high breast, colon, & lung cancer incidence rates **(B)**. Finally, the southeastern parts of the country exhibited the highest concentration of US counties with high SVI and high breast, colon, & lung cancer mortality rates **(C)**.

A total of 3,132 (99.7%) US counties were included in the analysis. The southwestern and southeastern parts of the country exhibited the highest concentration of US counties with high SVI and low breast & colon cancer screening rates (**Figure 1A**). By contrast, the southeastern parts of the country exhibited the highest concentration of US counties with high SVI and high breast, colon, & lung cancer incidence rates (**Figure 1B**). Finally, the southeastern parts of the country exhibited the highest concentration of US counties with high SVI and high breast, colon, & lung cancer mortality rates (**Figure 1C**). As expected, across the SVI tertiles, all four components of the SVI, namely socioeconomic status, household composition and disability, minority status and language, and housing type and transportation, exhibited a stepwise deterioration (**Supplementary Table 1**).

### 3.2 Impact of SVI on breast, colorectal, and lung cancer screening, incidence, and mortality rates

**Figure 2:**
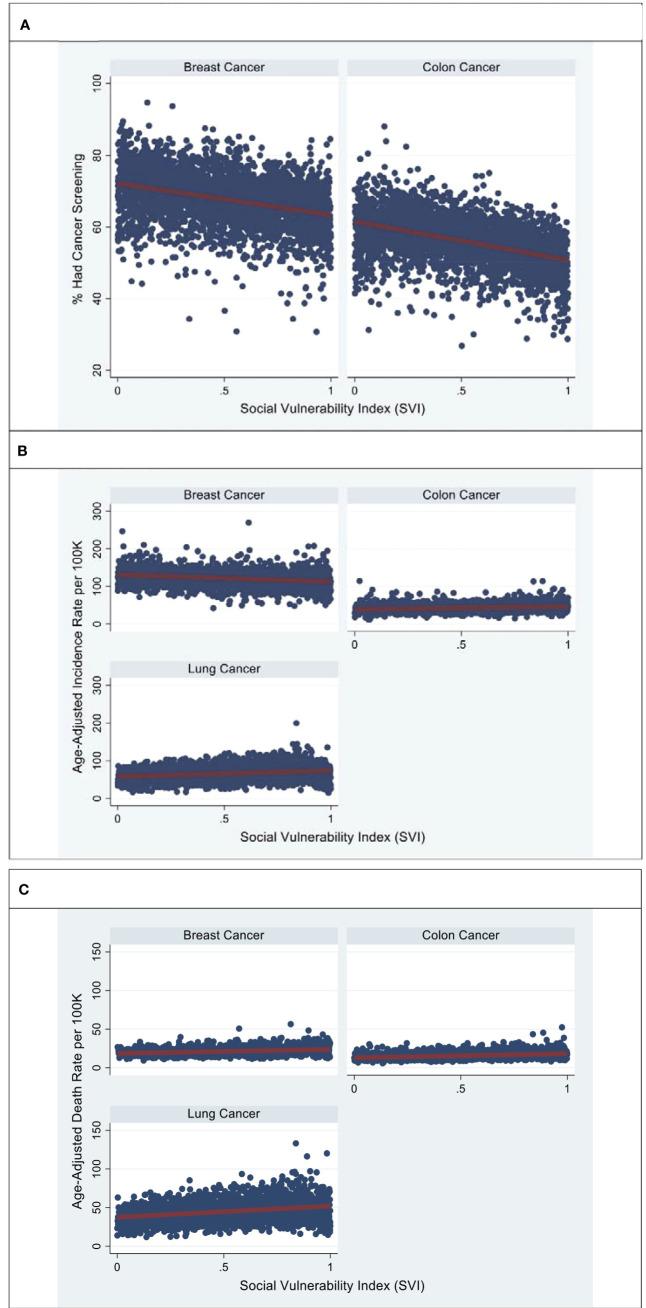
Represents age-adjusted breast, colon, and lung screening **(A)**, incidence **(B)**, and mortality rates **(C)**, by SVI. Source: Figures are based on the authors’ analysis of SVI and CDC cancer statistics data for breast, colon, and colon cancer. **(A)** County-level estimates of the percentage of individuals who had breast and colon screening across the SVI. **(B)** County-level estimates of the breast, colon, and lung cancer incidence rate across the SVI. **(C)** County-level estimates of breast, colon, and lung cancer death rates across the SVI. Models were adjusted for age.

Overall, breast and colorectal cancer screening rates demonstrated significant variation based on county SVI (**Table 1**). When comparing the lowest vs highest US county SVI tertiles, the impact of SVI on breast, colorectal, and lung cancer screening, incidence, and mortality rates were significant (**Table 1**).The percentage of women aged 40 years or older who had a mammogram in the past 2 years was lowest in individuals residing in the most socially vulnerable US counties (%: lowest SVI: 70.6 ± 6.93, intermediate SVI: 67.7 ± 6.76, highest SVI: 64.8 ± 6.99, p < 0.001) Similarly, the proportion of adults aged 50 years or older who had ever undergone colorectal endoscopy was lowest in individuals from the most socially vulnerable US counties (lowest SVI: 59.5 ± 6.55, intermediate SVI: 56.6 ± 6.14, highest SVI: 52.2 ± 6.27, p < 0.001). The relationship between age-adjusted incidence rates and SVI varied for breast, colorectal, and lung cancer. Specifically, the age-adjusted incidence rates (per 100,000 individuals) of breast cancer were lowest among individuals residing in the most socially vulnerable US counties (lowest SVI: 128 ± 19.9, intermediate SVI: 121 ± 19.3, highest SVI: 116 ± 20.5, p < 0.001). In contrast, age-adjusted incidence rates (per 100,000 individuals) of colorectal and lung cancer were highest among individuals residing in the most socially vulnerable US counties: colorectal (lowest SVI: 40.5 ± 9.93, intermediate SVI: 41.7 ± 8.17, highest SVI: 45.3 ± 10.4), lung (lowest SVI: 59.2 ± 14.8, intermediate SVI: 67.6 ± 16.7, highest SVI: 70.9 ± 19.7, p < 0.001). Among women with breast cancer, age-adjusted mortality rates (per 100,000 individuals) incrementally increased across SVI tertiles (lowest SVI: 19.7 ± 3.69, intermediate SVI: 20.8 ± 4.19, highest SVI: 23.0 ± 5.34, p < 0.001). Likewise, age-adjusted death rates (per 100,000 individuals) also significantly increased across SVI tertiles for patients with colorectal (lowest SVI: 14.0 ± 3.45, intermediate SVI: 15.3 ± 3.64, highest SVI: 17.4 ± 4.77, p < 0.001) and lung cancer (lowest SVI: 39.3 ± 9.91, intermediate SVI: 45.6 ± 11.6, highest SVI: 49.4 ± 14.1, p < 0.001). After confounder adjustment for age, SVI remained significantly associated with US county screening, incidence, and death rates per 100,000 individuals with breast, colorectal, and lung cancer (**Figures 2A–C**). For breast cancer, every 10-point increase in SVI was associated with a reduction in screening (-0.885, 95% CI -0.802 to -0.968), a reduction in incidence (-1.85, 95% CI -1.58 to -2.12), and an increase in mortality (0.525, 95% CI 0.448 to 0.602). In contrast, for colorectal cancer, every 10-point increase in SVI was associated with a reduction in screening (-0.885, 95% CI -0.802 to -0.968), an increase in incidence (-1.85, 95% CI -1.58 to -2.12), and an increase in mortality (0.525, 95% CI 0.448 to 0.602). Finally, for lung cancer, every 10-point increase in SVI was associated with an increase in incidence (1.61, 95% CI 1.37 to 1.84) and an increase in mortality (1.45, 95% CI 1.29 to 1.61).

**Table 1:** Cancer screening, incidence, and death rates across US counties by Social Vulnerability Index.

	Total (n = 3132)	1st Tertile (n = 1044)	2nd Tertile (n = 1044)	3rd Tertile (n = 1044)	p-values (highest vs lowest SVI)
Colorectal					
Had cancer screening	56.1 ± 6.99	59.5 ± 6.55	56.6 ± 6.14	52.2 ± 6.27	p < 0.001
Age-adjusted incidence rate per 100,000	42.7 ± 9.76	40.5 ± 9.93	41.7 ± 8.17	45.3 ± 10.4	p < 0.001
Age-adjusted death rate per 100,000	15.7 ± 4.27	14.0 ± 3.45	15.3 ± 3.64	17.4 ± 4.77	p < 0.001
Breast					
Had cancer screening	67.7 ± 7.29	70.6 ± 6.93	67.7 ± 6.76	64.8 ± 6.99	p < 0.001
Age-adjusted incidence rate per 100,000	121 ± 20.5	128 ± 19.9	121 ± 19.3	116 ± 20.5	p < 0.001
Age-adjusted death rate per 100,000	21.2 ± 4.69	19.7 ± 3.69	20.8 ± 4.19	23.0 ± 5.34	p < 0.001
Lung					
Had cancer screening (Data not available)					
Age-adjusted incidence rate per 100,000	66.4 ± 18.0	59.2 ± 14.8	67.6 ± 16.7	70.9 ± 19.7	p < 0.001
Age-adjusted death rate per 100,000	45.0 ± 12.7	39.3 ± 9.91	45.6 ± 11.6	49.4 ± 14.1	p < 0.001

### 3.3 Outcomes analysis

Our primary outcome analysis showed that US counties in the highest SVI tertile had lower odds of being in the ≥ 50th percentile for breast cancer screening (OR 0.241, 95% CI 0.201-0.290), lower odds of being in the ≥ 50th percentile for breast cancer incidence rates (OR 0.333, 95% CI 0.274-0.405), and higher odds of being in the ≥ 50th percentile for breast cancer mortality rates (OR 2.84, 95% CI 2.22-3.62) (**Table 2A**). With regards to colorectal cancers, US counties in the highest SVI tertile had lower odds of being in the ≥ 50th percentile for colorectal cancer screening (OR 0.153, 95% CI 0.126-0.185), higher odds of being in the ≥ 50th percentile for colorectal cancer incidence rates (OR 2.49, 95% CI 2.05-3.03), and higher odds of being in the ≥ 50th percentile for colorectal cancer mortality rates (OR 3.79, 95% CI 3.04-4.74) (**Table 2B**). Finally, US counties in the highest SVI tertile had higher odds of being in the ≥ 50th percentile for lung cancer incidence rates (OR 3.55, 95% CI 2.91-4.33) and higher odds of being in the ≥ 50th percentile for lung cancer mortality rates (OR 4.82, 95% CI 3.94-5.88) (**Table 2C**).

Table 2ALikelihood of being above US median screening, incidence, and death rate for breast cancer between most and least vulnerable US counties.

**Table:** 

Breast	Odds Ratio (95% Cl)
≥ US median screening rate	
SVI Tertile 1	Reference
SVI Tertile 2	0.496 (0.415 - 0.593)
SVI Tertile 3	0.241 (0.201 - 0.290)
≥ US median age-adjusted incidence rate per 100,000	
SVI Tertile 1	Reference
SVI Tertile 2	0.552 (0.455 - 0.670)
SVI Tertile 3	0.333 (0.274 - 0.405)
≥ US median age-adjusted death rate per 100,000	
SVI Tertile 1	Reference
SVI Tertile 2	1.65 (1.33 - 2.06)
SVI Tertile 3	2.84 (2.22 - 3.62)

**Table 2B:** Likelihood of being above US median screening, incidence, and death rate for colorectal cancer between most and least vulnerable US counties.

Colorectal	Odds Ratio (95% Cl)
≥ US median screening rate	
SVI Tertile 1	Reference
SVI Tertile 2	0.503 (0.420 - 0.603)
SVI Tertile 3	0.153 (0.126 - 0.185)
≥ US median age-adjusted incidence rate per 100,000	
SVI Tertile 1	Reference
SVI Tertile 2	1.47 (1.21 - 1.79)
SVI Tertile 3	2.49 (2.05 - 3.03)
≥ US median age-adjusted death rate per 100,000	
SVI Tertile 1	Reference
SVI Tertile 2	1.65 (1.33 - 2.06)
SVI Tertile 3	3.79 (3.04 - 4.74)

**Table 2C:** Likelihood of being above US median screening, incidence, and death rate for lung cancer between most and least vulnerable US counties.

Lung	Odds Ratio (95% Cl)
≥ US median screening rate	
SVI Tertile 1	Reference
SVI Tertile 2	Data Not Available
SVI Tertile 3	Data Not Available
≥ US median age-adjusted incidence rate per 100,000	
SVI Tertile 1	Reference
SVI Tertile 2	2.65 (2.18- 3.24)
SVI Tertile 3	3.55 (2.91 - 4.33)
≥ US median age-adjusted death rate per 100,000	
SVI Tertile 1	Reference
SVI Tertile 2	2.84 (2.33 - 3.45)
SVI Tertile 3	4.82 (3.94 - 5.88)

## 4 Discussion

### 4.1 SVI in current literature

The introduction of screening measures such as mammograms, colonoscopies, and low-dose computed tomography scans has significantly reduced cancer-related mortality. However, access to these life-saving screenings pose a major challenge. One study investigated the effect of a composite marker of SDOH, the Distressed Communities Index (DCI), on national breast and colorectal cancer screening, incidence, and mortality rates (31). However, the study had limitations in its focus on economic deprivation and limited social characteristics. Previous studies on cancer mortality and SDOH demonstrated cumulative increases in hazard ratios (HRs) with the presence of increasing numbers of SDOH factors (HR of 2.09, 95% CI 1.58-2.75 for those with 3 or more SDOHs) (32). Further, Coughlin et al. demonstrated that SDOH is correlated with breast cancer and colorectal cancer, specifically, age of diagnosis and survival outcomes (33, 34). Thus, such studies have noted several health disparities and significant differences in breast cancer mortality and stage at diagnosis based on specific SDOH factors. In the context of SVI, previous studies have highlighted the influence of SVI on health services utilization and outcomes, including COVID-19 incidence, mortality, obesity, surgery utilization, and cancer screening. The relevance of SVI in the spectrum of cancer care including diagnosis, treatment, recovery, and prevention have been previously studied on a wide variety of cancer types and varying outcome measures (35). For example, Bauer et al. evaluated the association between SVI and screening rates of breast, cervical and colorectal cancer based on the USPSTF guidelines and showed that the counties with highest SVI were associated with lower odds of cancer screening (36).

### 4.2 SVI and cancer outcomes

Our real-world findings validated the association of SVI with cancer diagnosis and outcomes. As SVI offers a broader perspective by considering factors such as household composition, disability, minority status, language, housing type, and transportation, this study is the first to investigate the relationship between social vulnerability, as measured by the SVI, and breast, colorectal, and lung cancer screening, incidence, and mortality rates in US counties. Overall, lower breast and colorectal cancer screening rates correlated with high SVI scores. In addition, higher colorectal and lung cancer incidence rates were seen in counties with high SVI scores. Interestingly, there was lower breast cancer incidence in counties with higher SVI due to other factors not evaluated in this dataset. Further, age-adjusted death rates for all three cancer types (breast, colorectal and lung) increased with increasing SVI scores and across SVI tertiles. These findings highlight the correlation between SDOH/health disparities and cancer outcomes, with adverse cancer outcomes in areas with high social vulnerability.

In addition, this study extends prior work by demonstrating a strong inverse correlation between US county SVI and the likelihood of being in the ≥ 50th percentile for breast and colorectal cancer screening. Furthermore, our findings also indicate a strong positive correlation between US county SVI and the likelihood of being in the ≥ 50th percentile for colorectal and lung cancer incidence and mortality.

These findings have important implications for health policy reform aimed at reducing disparities in cancer care continuum. These results underscore the need to incorporate measures like SVI in the design and distribution of targeted cancer screening programs, such as mobile screening service facilities and extended hour screening clinics, to ensure equitable access for marginalized communities across the SDOH spectrum. In addition, there is a need for addressing the structural inequality that exists in the US and seeking systematic ways to extend cancer care, diagnosis, prevention, and treatment for those living in counties with high SVI.

### 4.3 Limitations

That said, this study has limitations. First, the cross-sectional design prevents establishing a causal relationship or demonstrating a clear longitudinal association between SVI and cancer screening, incidence, and mortality rates. Further, given the ecological nature of the study with data from the BRFSS and NHIS which provide aggregated county-level estimates rather than individual rates, the study is limited in its ability to make inferences at the individual level aggregate data. As such, direct implications on an individual or single-county level cannot be made. However, the findings highlight existing cancer disparities and are meaningful. In addition, as this study utilizes publicly available data reported by the CDC/ATSDR, the years encompassed in the study are based on currently available data. However, the data offers insight into trends of cancer care and outcomes. As such, future studies to further validate the findings and analyze the latest available data are recommended. Additionally, while SVI captures a broad range of SDOH compared to other indices like ADI, it still does not encompass all relevant social elements, such as food insecurity and healthcare access.

## 5 Conclusions

The significance of our findings goes beyond establishing an association between social vulnerability, as measured by SVI, and breast, colorectal, and lung cancer screening, incidence, and mortality rates in US counties. Since SVI provides a comprehensive measure of SDOH, it is crucial for public health and policy experts to consider its use in identifying marginalized communities that would benefit from targeted programs aimed at improving cancer screening rates and healthcare access. Future research should focus on studies to better understand the longitudinal correlation between SVI and cancer screening, incidence, and mortality rates.

## Data availability statement

Publicly available datasets were analyzed in this study. This data can be found here: https://www.atsdr.cdc.gov/placeandhealth/svi/documentation/svi_documentation_2018.html.

## Ethics statement

Ethical approval was not required for the study involving humans in accordance with the local legislation and institutional requirements. Written informed consent to participate in this study was not required from the participants or the participants’ legal guardians/next of kin in accordance with the national legislation and the institutional requirements.

## Author contributions

AM: Conceptualization, Data curation, Methodology, Software, Writing – original draft, Writing – review & editing. WJ: Visualization, Writing – original draft, Writing – review & editing. GN: Writing – original draft, Writing – review & editing, Conceptualization, Supervision.

## References

[1] Murphy SL Kochanek KD Xu JQ Arias E. Mortality in the United States, 2020. NCHS Data Brief, no 427. Hyattsville, MD: National Center for Health Statistics (2021). doi: 10.15620/cdc:112079 34978528

[2] Goding Sauer A Siegel RL Jemal A Fedewa SA. Current prevalence of major cancer risk factors and screening test use in the United States: disparities by education and race/ethnicity. Cancer Epidemiol Biomarkers Prev. (2019) 28:629–42. doi: 10.1158/1055-9965.EPI-18-1169 30944145

[3] National Cancer Institute. Cancer Disparities . Available online at: https://www.cancer.gov/about-cancer/understanding/disparities (Accessed 16 April 2024).

[4] Zeng C Wen W Morgans AK Pao W Shu XO Zheng W. Disparities by race, age, and sex in the improvement of survival for major cancers: results from the national cancer institute surveillance, epidemiology, and end results (SEER) program in the United States, 1990 to 2010. JAMA Oncol. (2015) 1:88–96. doi: 10.1001/jamaoncol.2014.161 26182310 PMC4523124

[5] American Cancer Society. Cancer Facts & Figures 2022 (2022). Atlanta: American Cancer Society. Available online at: https://www.cancer.org/content/dam/cancer-org/research/cancer-facts-and-statistics/annual-cancer-facts-and-figures/2022/2022-cancer-facts-and-figures.pdf (Accessed 16 April 2024).

[6] Centers for Disease Control and Prevention. Health, United States, 2020 – Data Finder . Available online at: https://www.cdc.gov/nchs/hus/data-finder.htm (Accessed 16 April 2024).

[7] U.S. Cancer Statistics Working Group. U.S. Cancer Statistics Data Visualizations Tool, based on 2021 submission data (1999-2019) (2022). U.S. Department of Health and Human Services, Centers for Disease Control and Prevention and National Cancer Institute. Available online at: https://www.cdc.gov/cancer/dataviz (Accessed 16 April 2024).

[8] Singh GK Jemal A. Socioeconomic and racial/ethnic disparities in cancer mortality, incidence, and survival in the United States, 1950–2014: over six decades of changing patterns and Widening inequalities. J Environ Public Health. (2017) 2017:2819372. doi: 10.1155/2017/2819372 28408935 PMC5376950

[9] Centers for Disease Control and Prevention. Agency for Toxic Substances and Disease Registry/ Geospatial Research, Analysis, and Services Program. CDC/ATSDR Social Vulnerability Index (2018). Available at: https://www.atsdr.cdc.gov/placeandhealth/svi/documentation/SVI_documentation_2018.html.

[10] Neelon B Mutiso F Mueller NT Pearce JL Benjamin-Neelon SE. Spatial and temporal trends in social vulnerability and COVID-19 incidence and death rates in the United States. PloS One. (2021) 16:e0248702. doi: 10.1371/journal.pone.0248702 33760849 PMC7990180

[11] Diaz A Hyer JM Barmash E Azap R Paredes AZ Pawlik TM. County-level social vulnerability is associated with worse surgical outcomes especially among minority patients. Ann Surg. (2021) 274:881. doi: 10.1097/SLA.0000000000004691 33351455

[12] Biggs EN Maloney PM Rung AL Peters ES Robinson WT. The relationship between social vulnerability and COVID-19 incidence among louisiana census tracts. Front Public Health. (2021) 8:617976. doi: 10.3389/fpubh.2020.617976 33553098 PMC7856141

[13] Yu CY Woo A Emrich CT Wang B. Social Vulnerability Index and obesity: An empirical study in the US. CITIES. (2020) 97. doi: 10.1016/j.cities.2019.102531

[14] Akinlotan MA Weston C Bolin JN. Individual- and county-level predictors of cervical cancer screening: a multi-level analysis. Public Health. (2018) 160:116–24. doi: 10.1016/j.puhe.2018.03.026 29803186

[15] Chan DNS Law BMH Au DWH So WKW Fan N. A systematic review of the barriers and facilitators influencing the cancer screening behaviour among people with intellectual disabilities. Cancer Epidemiol. (2022) 76:102084. doi: 10.1016/j.canep.2021.102084 34920342

[16] Coughlin SS King J Richards TB Ekwueme DU. Cervical cancer screening among women in metropolitan areas of the United States by individual-level and area-based measures of socioeconomic status, 2000 to 2002. Cancer Epidemiology Biomarkers Prev. (2006) 15:2154–9. doi: 10.1158/1055-9965.EPI-05-0914 17119040

[17] Coughlin SS Leadbetter S Richards T Sabatino SA. Contextual analysis of breast and cervical cancer screening and factors associated with health care access among United States women, 2002. Soc Sci Med. (2008) 66:260–75. doi: 10.1016/j.socscimed.2007.09.009 18022299

[18] Pruitt SL Shim MJ Mullen PD Vernon SW Amick BC. The association of area socioeconomic status and breast, cervical, and colorectal cancer screening: A systematic review. Cancer Epidemiol Biomarkers Prev. (2009) 18:2579–99. doi: 10.1158/1055-9965.EPI-09-0135 PMC2760038 19815634

[19] Ramjan L Cotton A Algoso M Peters K. Barriers to breast and cervical cancer screening for women with physical disability: A review. Women Health. (2016) 56:141–56. doi: 10.1080/03630242.2015.1086463 26325597

[20] Warren Andersen S Blot WJ Lipworth L Steinwandel M Murff HJ Zheng W. Association of race and socioeconomic status with colorectal cancer screening, colorectal cancer risk, and mortality in southern US adults. JAMA Netw Open. (2019) 2:e1917995. doi: 10.1001/jamanetworkopen.2019.17995 31860105 PMC6991213

[21] State Cancer Profiles. Available online at: https://www.statecancerprofiles.cancer.gov/index.html (Accessed 16 April 2024).

[22] State Cancer Profiles. Data Types—Screening and Risk Factors Data Types (2017). Available online at: https://statecancerprofiles.cancer.gov/screening_risk_datatypes.html (Accessed 16 April 2024).

[23] Liu B Parsons V Feuer EJ Pan Q Town M Raghunathan TE. Small area estimation of cancer risk factors and screening behaviors in US counties by combining two large national health surveys. Prev Chronic Dis. (2019) 16:E119. doi: 10.5888/pcd16.190013 31469068 PMC6716412

[24] National Institutes of Health National Cancer Institute. Methodology for the model-based small area estimates of cancer risk factors and screening behaviors. Small Area Estimates Cancer-Related Measures.

[25] National Institutes of Health National Cancer Institute. State Cancer Profiles. Breast cancer incidence rates table. Available online at: https://www.statecancerprofiles.cancer.gov/incidencerates/index.php?stateFIPS=00&areatype=county&cancer=055&race=00&sex=2&age=001&stage=999&year=0&type=incd&sortVariableName=rate&sortOrder=default&output=0#results (Accessed 16 April 2024).

[26] National Institutes of Health National Cancer Institute. State Cancer Profiles. Colon and rectal cancer incidence rates table. Available online at: https://www.statecancerprofiles.cancer.gov/incidencerates/index.php?stateFIPS=00&areatype=county&cancer=020&race=00&sex=0&age=001&stage=999&year=0&type=incd&sortVariableName=rate&sortOrder=default&output=0#results (Accessed 16 April 2024).

[27] National Institutes of Health National Cancer Institute. State Cancer Profiles. Lung cancer incidence rates table. Available online at: https://www.statecancerprofiles.cancer.gov/incidencerates/index.php?stateFIPS=00&areatype=county&cancer=047&race=00&sex=0&age=001&stage=999&year=0&type=incd&sortVariableName=rate&sortOrder=default&output=0#results (Accessed 16 April 2024).

[28] National Institutes of Health National Cancer Institute. State Cancer Profiles. Breast cancer death rates table. Available online at: https://www.statecancerprofiles.cancer.gov/deathrates/index.php?stateFIPS=00&areatype=county&cancer=055&race=00&sex=2&age=001&year=0&type=death&sortVariableName=rate&sortOrder=default#results (Accessed 16 April 2024).

[29] National Institutes of Health National Cancer Institute. State Cancer Profiles. Colon and rectal cancer death rates table. Available online at: https://www.statecancerprofiles.cancer.gov/deathrates/index.php?stateFIPS=00&areatype=county&cancer=020&race=00&sex=0&age=001&year=0&type=death&sortVariableName=rate&sortOrder=default#results (Accessed 16 April 2024).

[30] National Institutes of Health National Cancer Institute. State Cancer Profiles. Lung cancer death rates table. Available online at: https://www.statecancerprofiles.cancer.gov/deathrates/index.php?stateFIPS=00&areatype=county&cancer=047&race=00&sex=0&age=001&year=0&type=death&sortVariableName=rate&sortOrder=default#results (Accessed 16 April 2024).

[31] Herbert C Paro A Diaz A Pawlik TM. Association of community economic distress and breast and colorectal cancer screening, incidence, and mortality rates among US counties. Ann Surg Oncol. (2022) 29:837–48. doi: 10.1245/s10434-021-10849-7 34585297

[32] Pinheiro LC Reshetnyak E Akinyemiju T Phillips E Safford MM. Social Determinants of Health and Cancer Mortality in the REasons for Geographic and Racial differences in Stroke (REGARDS) cohort study. Cancer. (2022) 128:122–30. doi: 10.1002/cncr.33894 PMC9301452 34478162

[33] Coughlin SS. Social determinants of colorectal cancer risk, stage, and survival: a systematic review. Int J Colorectal Dis. (2020) 35:985–95. doi: 10.1007/s00384-020-03585-z 32314192

[34] Coughlin SS. Social determinants of breast cancer risk, stage, and survival. Breast Cancer Res Treat. (2019) 177:537–48. doi: 10.1007/s10549-019-05340-7 31270761

[35] Tran T Rousseau MA Farris DP Bauer C Nelson KC Doan HQ. The social vulnerability index as a risk stratification tool for health disparity research in cancer patients: a scoping review. Cancer Causes Control. (2023) 34:407–20. doi: 10.1007/s10552-023-01683-1 PMC10080510 37027053

[36] Bauer C Zhang K Xiao Q Lu J Hong YR Suk R. County-level social vulnerability and breast, cervical, and colorectal cancer screening rates in the US, 2018. JAMA Netw Open. (2022) 5:e2233429. doi: 10.1001/jamanetworkopen.2022.33429 36166230 PMC9516325

