# Supplementary material for: Association of US county-level social vulnerability index with breast, colorectal, and lung cancer screening, incidence, and mortality rates across US counties
Source: Front Oncol. 2024 Aug 7;14:1422475. doi: 10.3389/fonc.2024.1422475 (PMC11335618; doi:10.3389/fonc.2024.1422475)
Supplement: Supplementary file 1 [file Table_1.docx]

| **Supplemental Table 1.** Socioeconomic Status, Household Composition & Disability, Minority Status & Language, Housing Type & Transportation across US counties by Social Vulnerability Index. | | | | | |
| --- | --- | --- | --- | --- | --- |
|  | **Total**  **(n = 3132)** | **1st Tertile**  **(n = 1044)** | **2nd Tertile**  **(n = 1044)** | **3rd Tertile**  **(n = 1044)** | **p-values (highest vs lowest SVI)** |
| Socioeconomic Status |  |  |  |  |  |
| Percentage of persons below poverty estimate | 15.6 ± 6.44 | 10.2 ± 3.15 | 15.0 ± 3.76 | 21.5 ± 5.99 | p < 0.001 |
| Unemployment Rate estimate % | 5.76 ± 2.81 | 3.77 ± 1.57 | 5.64 ± 1.80 | 7.87 ± 3.09 | p < 0.001 |
| Per capita income estimate, 2014-2018 ACS, % | 27,028 ± 6491 | 31,911 ± 6229 | 26,945 ± 5135 | 22,228 ± 3792 | p < 0.001 |
| Percentage of persons with no high school diploma (age 25±} estimate, % | 13.4 ± 6.34 | 8.23 ± 2.95 | 13.0 ± 4.42 | 19.0 ± 5.86 | p < 0.001 |
| Percentage uninsured in the total civilian noninstitutionalized population estimate, 2014-2018 ACS, % | 10.1 ± 5.06 | 7 .14 ± 3.25 | 9.89 ± 4.50 | 13.1 ± 5.30 | p < 0.001 |
| Household Composition & Disability |  |  |  |  |  |
| Percentage of persons aged 65 and older estimate, 2014-2018 ACS | 18.4 ± 4.58 | 19.3 ± 4.94 | 18.6 ± 4.66 | 17.3 ±3.84 |  |
| Percentage of persons aged 17 and younger estimate, 2014-2018 ACS | 22.4 ± 3.46 | 21.9 ± 3.28 | 21.9 ± 3.33 | 23.3 ± 3.56 | p < 0.001 |
| Percentage of civilian noninstitutionalized population with a disability estimate, 2014-2018 ACS | 15.9 ± 4.41 | 13.3 ± 3.15 | 16.2 ± 4.00 | 18.2 ± 4.49 | p < 0.001 |
| Percentage of single parent households with children under 18 estimate, 2014-2018 ACS | 8.31 ± 2.73 | 6.63 ± 1.86 | 7.99 ± 2.01 | 10.3 ± 2.82 | p < 0.001 |
| Minority Status & Language |  |  |  |  | p < 0.001 |
| Percentage minority {all persons except white, non-Hispanic) estimate, 2014-2018 ACS | 23.4 ± 20.1 | 11.6 ± 9.62 | 19.6 ± 15.3 | 39.0 ± 22.0 |  |
| Percentage of persons (age 5+} who speak English "less than well" estimate, 2014-2018 ACS | 1.70 ± 2.79 | 0.845 ± 1.15 | 1.61 ± 2.23 | 2.65 ± 3.92 | p < 0.001 |
| Housing Type & Transportation |  |  |  |  |  |
| Percentage of housing in structures with 10 or more units estimate | 4.68 ± 5.71 | 5.10 ± 5.67 | 5.20 ± 6.44 | 3.76 ± 4.80 | p < 0.001 |
| Percentage of mobile homes estimate | 12.9 ± 9.61 | 7 .30 ± 5.51 | 12.4 ± 7.94 | 19.1 ± 10.7 | p < 0.001 |
| Percentage of occupied housing units with more people than rooms estimate | 2.40 ± 2.22 | 1.42 ± 0.956 | 2.25 ± 1.44 | 3.53 ± 3.09 | p < 0.001 |
| Percentage of households with no vehicle available estimate | 6.30 ± 4.20 | 4.33 ± 1.84 | 6.26 ± 3.76 | 8.32 ± 5.24 | p < 0.001 |
| Percentage of persons in group quarters estimate, 2014-2018 ACS | 3.52 ± 4.57 | 2.50 ± 3.06 | 3.57 ± 4.45 | 4.50 ± 5.62 | p < 0.001 |
|  |  |  |  |  |  |
